# Recent Levels of Technetium-99 in Seawater at the West Coast of Svalbard

**DOI:** 10.1100/tsw.2002.291

**Published:** 2002-05-31

**Authors:** Sebastian Gerland, Bjørn Lind, Mark Dowdall, Anne Kathrine Kolstad

**Affiliations:** Norwegian Radiation Protection Authority, Environmental Unit, Tromsø, Norway; Norwegian Radiation Protection Authority, Østerå s, Norway, Norway

**Keywords:** radionuclides, radioecology, oceanography, Arctic, Svalbard, technetium

## Abstract

Seawater from the western coast of Svalbard was sampled in the spring and summer of 2000 to determine levels of technetium-99 (^99^Tc), a conservative-behaving, manmade radionuclide originating from European nuclear reprocessing plants. This paper deals with the recent levels of this radionuclide in seawater and with the link between an Arctic fjord, Kongsfjorden, and the Western Spitsbergen Current (WSC), investigated using ^99^Tc results. By means of the WSC, the ^99^Tc radionuclides ultimately reach the eastern Fram Strait west of Spitsbergen (the largest island of the Svalbard archipelago). Results from oceanographic modelling and sea ice observations indicate a direct coupling between Kongsfjorden and the area west of it. The findings in connection with new radionuclide results presented in this paper concur with these assumptions. Furthermore they indicate that the inner part of Kongsfjorden is also well linked to the WSC. Surface seawater from the central part of the WSC, sampled during a cruise with RV Polarstern in the summer of 2000, shows a higher level of ^99^Tc than those measured in Kongsfjorden in spring 2000. However, all levels measured in surface water are of the same order of magnitude. Data from sampling of deeper water in the WSC area provide information pertaining to the lateral distribution of ^99^Tc. The results, along with additional data from spring 2001, indicate that Kongsfjorden is suitable for monitoring the levels of ^99^Tc arriving in the European Arctic and that the sheltered setting of this fjord does not necessarily provide protection against pollution from the open sea.

## INTRODUCTION

Radionuclides, both artificial and natural, from various sources, are transported over long ranges by ocean currents and are an important issue within environmental pollution studies and monitoring[1], as well as within oceanography.

The anthropogenic, highly soluble, and long-lived radionuclide technetium-99 ($^{99}$Tc, half-life: 213,000 years) was, and is, discharged into the seas of central Europe by nuclear reprocessing plants such as Sellafield (U.K.) and La Hague (France). $^{99}$Tc is a soft β-radiation emitter, and its presence along the Norwegian Coast and further north after enhanced discharges in the mid-1990s is documented in a number of publications[2,3,4]. The global background of $^{99}$Tc in the environment originates from earlier releases from nuclear installations, from fallout in connection with atmospheric nuclear weapon tests, and from the use of $^{99m}$Tc in diagnostic nuclear medicine[5]. Dahlgaard[6] provides a portioning of sources of $^{99}$Tc in seawater in the East Greenland Current over the period 1988–1990 of about 85% from European coastal discharges and 15% from nuclear weapon tests. The Norwegian Radiation Protection Authority (NRPA) commenced an investigation into long-range transported radionuclides off the western coast of Spitsbergen in the spring of 2000[4], with a sampling program in Kongsfjorden, an Arctic fjord[7,8]. Further samples were collected on a cruise with the ice-breaking research vessel *Polarstern* in the summer of 2000 and again in Kongsfjorden in May 2001.

The transport of radionuclide discharges from European nuclear reprocessing plants was studied in detail in the 1970s and 1980s, when enhanced amounts of caesium-137 were discharged[9,10]. These discharges could be detected as far north as the Fram Strait and the Arctic Ocean. Dahlgaard[9] reported a corresponding transfer time of 4 years from the reprocessing plant at Sellafield, i.e., from the Irish Sea, to the Fram Strait.

Although the amount of new samples in this study is very limited and the samples were not taken in the same month, we find that the results give some valuable indications that are useful in further radioecological and oceanographic studies, to improve the understanding of both the West Spitsbergen Current (WSC)[11] and the Kongsfjorden environments. However, this study is meant to be the beginning of a period of long-term observation and monitoring to study the processes leading to the observed radionuclide levels in detail.

## SAMPLES, PROCESSING, AND MEASUREMENTS

Seawater samples of 50 l were obtained in Kongsfjorden in May 2000 during NRPA's Svalbard-2000 (station codes beginning with SV00) field campaign[4], in spring 2001 (station codes beginning with SV01), and on a cruise to the Fram Strait (station codes beginning with PS) with the RV *Polarstern*[12]. At each of the two stations off Kongsfjorden (PS173 and PS264), three samples were taken (respectively, surface, subsurface, and deepwater). The water sample PS 264-2 was sampled at 100 m below the surface (see Table 1), and the corresponding CTD profile (Fig. 1, pers. comm. U. Schauer 2001[13]), measured before the sampling, indicates due to its salinity maximum the presence of the WSC. The sampling depth for PS 173-3 (152 m) was chosen according to a CTD profile taken prior to the sampling. Deepwater samples were obtained at 1,000 m (PS173-1) and at 465 m (PS264-2). However, the sampling in the fjord was logistically limited to surface sampling. Two more samples were taken in Kongsfjorden in May 2001, one at an identical spot as in 2000, the other one in the northern part of inner Kongsfjorden. The results from this sampling give the possibility to compare levels from two consecutive years, and they reduce somewhat the problem with interpretation limits due to the very limited number of samples.

The 50-l seawater samples were pumped through a 1-µm polypropylene filter to remove suspended matter. $^{99m}$Tc is used as a tracer to quantify the effectiveness of the radionuclide separation. In the premeasurement sample processing, the $^{99}$Tc radionuclides are separated from other nuclides

**TABLE 1**
**Sample Location Coordinates for Seawater Sampling, Station ID, Sampling Depth and Date, and $^{99}$Tc Results with Associated Errors (Station Order from East to West)**

| Latitude | Longitude | Station ID | Sampling Depth (m) | Date | $^{99}$Tc in Seawater (Bq m$^{-3}$) |
|---|---|---|---|---|---|
| *N 78° 58.092* | *E 12° 15.089* | *SV01-11b* | *Surface* | *28-05-01* | *0.21 ± 0.04* |
| N 78° 54.973 | E 12° 13.389 | SV00-2a | Surface | 17-05-00 | 0.21 ± 0.03 |
| N 78° 55.711 | E 11° 56.313 | SV00-4b | Surface | 19-05-00 | 0.25 ± 0.03 |
| *N 78 55.711* | *E 11 56.313* | *SV01-9b* | *Surface* | *24-05-01* | *0.20 ± 0.03* |
| N 78° 59.505 | E 11° 43.478 | SV00-1b | Surface | 16-05-00 | 0.26 ± 0.03 |
| N 78° 55.2 | E 8° 28.1 | PS 264-3 | Surface | 18-08-00 | 0.39 ± 0.05 |
| N 78° 55.2 | E 8° 28.1 | PS 264-2 | 100 | 18-08-00 | 0.27 ± 0.03 |
| N 78° 55.2 | E 8° 28.1 | PS 264-2 | 465 | 18-08-00 | 0.17 ± 0.03 |
| N 78° 50.055 | E 4° 2.445 | PS 173-3a | Surface | 04-08-00 | 0.22 ± 0.03 |
| N 78 50.055 | E 4 2.445 | PS 173-3 | 152 | 04-08-00 | 0.14 ± 0.03 |
| N 78° 50.055 | E 4° 2.445 | PS 173-1 | 1000 | 04-08-00 | 0.04 ± 0.02 |

*Note:* The two data points from 2001 are printed in italics. Nonsurface samples are given in light blue (subsurface water) and darker blue (deep water).

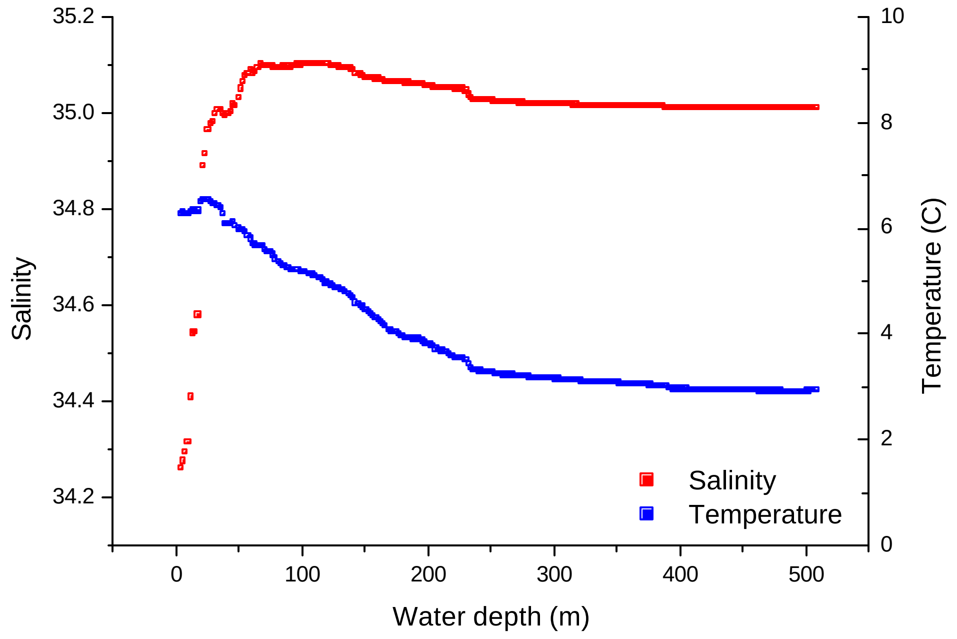

**FIGURE 1.** CTD (Conductivity-Temperature-Depth) profile from station PS 264. Unpublished data by Schauer et al.[13]. In addition to surface sampling, the water sampling depths were chosen at 100 and 465 m, because the maximum in salinity indicates that Atlantic water from the West Spitsbergen Current (WSC) and near the bottom, deepwater can be expected.

by ion-exchange chromatography using AG1-X4 resin (100- to 200-µm mesh size, BIO-RAD Laboratories, Hercules, CA) and subsequent separation techniques such as coprecipitation and solvent extraction. Finally, the technetium isotopes are electroplated onto steel disks. The yield of the $^{99m}$Tc tracer (typically 70–80%) on the disks is counted on a NaI well detector (Canberra, CT). One week later, the $^{99}$Tc activity on the disks is measured with a low-background, anticoincidence β-counter (type GM-25-5, RISØ, Roskilde, Denmark). The analytical set up allows for four parallel sample measurements. The results for seawater have an error of 8% (for seaweed 5%)[14]. The method for measuring $^{99}$Tc in seawater and in biota is described in detail by Kolstad et al.[15] and Chen et al.[16].

## RESULTS

The five sample locations (Fig. 2) form a profile from the deep sea (station PS173, water depth: 2,298 m) via the shelf edge (station PS 264, water depth: 509 m) to the outer (station SV00-1b), central (station SV00-4b, water depth <10 m), and inner Kongsfjorden (station SV00-2a). Exact water depths for the Kongsfjorden stations are unavailable.

As one would expect, the levels of $^{99}$Tc were highest in the centre of the WSC with 0.39 Bq/m$^3$, measured on the surface water sample. The measurement at the deep-sea location farthest west, with 0.22 Bq/m$^3$ only approximately 55% of the central WSC level, indicates that the centre of the WSC is fairly narrow and does not extend with the same water composition very far west, as oceanographers have concluded from detailed CTD studies[13]. Haugan[11] measured the highest water salinities (>35.05) and temperatures (>4°C) over the continental slope west of Spitsbergen, but not further west above deeper water (June 1998). East towards the coast and Kongsfjorden, the levels in

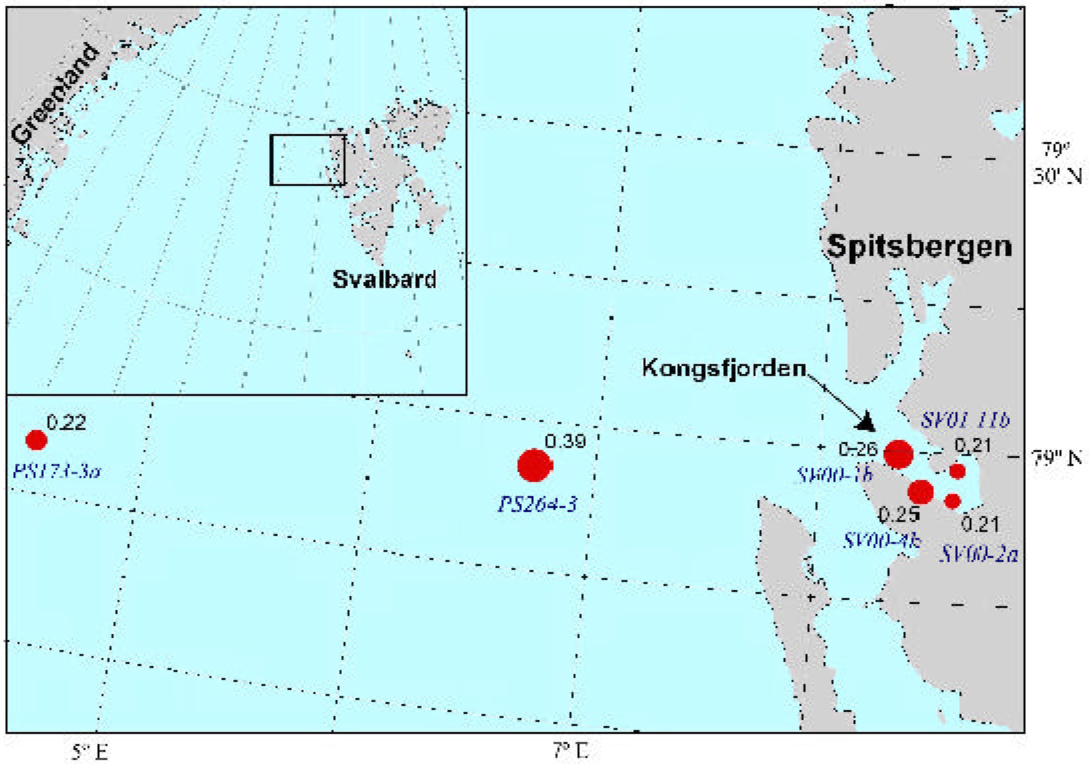

**FIGURE 2.** Map of the eastern part of the Fram Strait with Kongsfjorden. Seawater $^{99}$Tc data from surfacewater sampling are indicated with circles, the levels are marked by the circles size and numbers in Bq/m$^3$.

the surface water remain similar or higher (between 0.26 and 0.21 Bq/$m^3$ in the fjord) than at the deep-sea location (PS173) in the west. This indicates that the Kongsfjorden is substantially influenced by the Atlantic water from the WSC.

Results from below-surface water and deepwater sampling from stations PS173 and PS264 show a decrease of $^{99}$Tc with depth for both stations (Fig. 3, Table 1). This scenario concurs with earlier observations in the East Greenland Current made by Dahlgaard[6], who also found the highest concentrations of $^{99}$Tc in the surface or near-surface layers. Since the discharges are released near the surface and $^{99}$Tc is behaving conservatively, it is not surprising to find the highest levels in the upper part of the water column (pers. comm. U. Schauer 2002). At the deep-sea location (PS173), the levels decrease more with sampling depth than at the shelf edge (PS264), where even at the deep sampling spot the level is, at 0.17 Bq/$m^3$, more than 40% of the surface $^{99}$Tc level at the same location. Correspondingly at the deepwater location, the level at 1,000 m depth is, at 0.04 Bq/$m^3$, only 18% of that of the surface value. The below-surface water sampling on the shelf edge position (PS264) provided a value of 0.27 Bq/$m^3$. This is 69% of the surface water value at the same geographic position. At the deep-sea position (PS173), the level of $^{99}$Tc was 0.14 Bq/$m^3$ (63% of the surface water value at that position). Vertical profiling of $^{137}$Cs measurements of seawater in the Norwegian Sea and the Fram Strait in 1995, also in connection with discharges from Sellafield, also showed a decrease of levels with depth[17,18]. Two additional results from sampling in Kongsfjorden at the same location as for SV00-4b from 2001 (SV01-9b) and in the northeastern part of inner Kongsfjorden (SV01-11b) agree with 0.20 and 0.21 Bq/$m^3$, respectively (see Table 1).

## DISCUSSION

When reviewing the recent $^{99}$Tc results in a broader context, both spatially and temporally, the levels west of Svalbard are approximately six to seven times higher than those of surface water sampled in the same area in the mid-1990s[19], before the enhanced $^{99}$Tc discharges from Sellafield could have

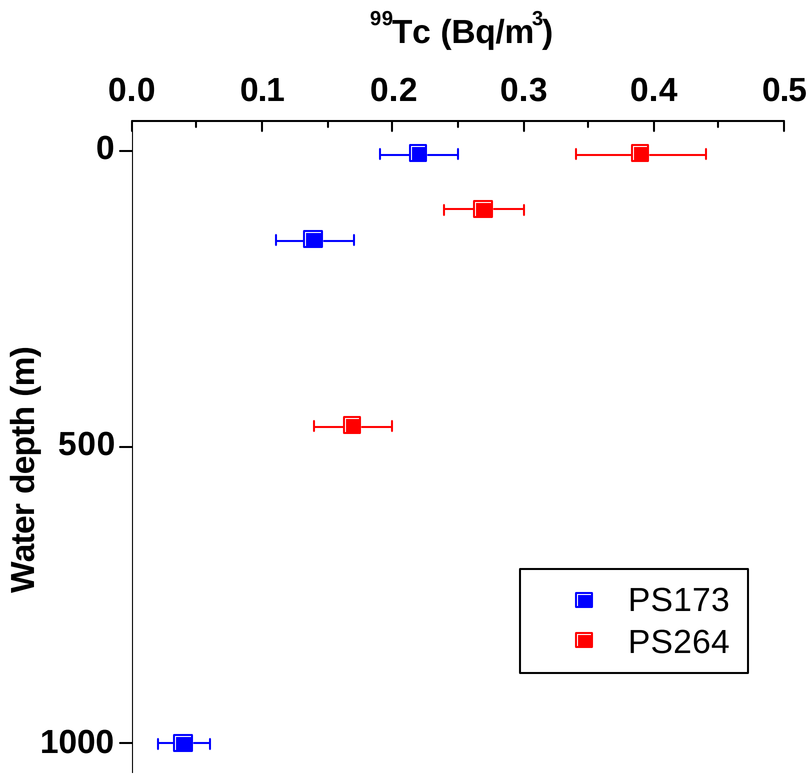

**FIGURE 3.** $^{99}$Tc data from seawater at the locations off Kongsfjorden vs. sampling depth.

reached Svalbard. This substantial increase in the $^{99}Tc$ concentration in the marine environment west of Svalbard is likely caused by enhanced discharges at the nuclear reprocessing plant at Sellafield. However, levels measured at Hillesøy on the coast of northern Norway, also in 2000[5], are again five times higher than in Kongsfjorden at the same time. $^{99}Tc$ data obtained from seaweed samples from Kongsfjorden in May 2000 (*Fucus distichus*) with a level of 34.3 Bq/kg[4] confirm the enhanced $^{99}Tc$ levels as measured in the seawater, when assuming similar concentration factors as those found for similar *Fucus* species.

Although the data presented in this paper are sparse, the amount and results provide significant insight into the coupling of the WSC to the inner part of Kongsfjorden. Ingvaldsen and others[20] comment that the water exchange between the shelf area west of Spitsbergen and Kongsfjorden seems strong. Water temperature and sea-ice thickness monitoring also indicate substantial warmwater influence in the northern part of inner Kongsfjorden (Dyrevika) in winter, resulting in reduced sea-ice growth[21]. This effect of ocean heat flux and correspondingly relatively light ice conditions in Spitsbergen fjords lacking a shallow sill is also mentioned by Saloranta and Svendsen[22] as an indication of the influence of Atlantic water from the WSC reaching the shelf and fjords. Our $^{99}Tc$ results, presented in this paper, strengthen these statements and indicate, in addition, that the inner part of Kongsfjorden is also influenced by water exchange processes, where we could detect $^{99}Tc$ levels slightly lower than in the outer part of Kongsfjorden.

The link between the Atlantic water of the WSC and Kongsfjorden makes this fjord, along with the existing infrastructure in Ny-Ålesund, a suitable location for regular radioecological and tracer monitoring of seawater. In the spring of 2001, the NRPA commenced a monthly monitoring program for $^{99}Tc$ in seawater at Ny-Ålesund, as part of the Norwegian marine surveillance program. Along with sampling at Bjørnøya, Hopen, Jan Mayen, and Hillesøy, these data are intended to resolve, along with numerical oceanographic models, the complex transport processes of this radionuclide to the Arctic. Then, having a larger database, we anticipate being able to validate our conclusions from this start-up study. Furthermore, with these data, seasonally changing processes that are typical for these environments (e.g., sea-ice formation and melting) and their influence on the oceanographic conditions can be studied in more detail. The results from the two locations off Kongsfjorden with sampling at different depths show the possibilities of how radioecology can support oceanographic studies. However, for the provision of substantial input, more data are needed both in the spatial and temporal dimensions.

## ACKNOWLEDGEMENTS

We are grateful to the captain, crew, and scientific cruise leader U. Schauer (Alfred Wegener Institute for Polar and Marine Research, Bremerhaven, Germany) onboard the RV *Polarstern* for help during the cruise ARK-XVI/2 in 2000 and for discussions regarding the oceanography in the Fram Strait. We thank S. Grøttheim for taking part in the sampling work on board the RV *Polarstern*. J. Drefvelin (NRPA) assisted with producing the map in Fig. 2. We are grateful to the CTD team onboard RV *Polarstern*, especially to P. Eriksson, U. Schauer, and H. Witte. U. Schauer kindly provided us with the CTD data from station PS 264-1 and helped with discussions. Constructive criticism from C. Papucci (ENEA, Italy) and a second anonymous reviewer is gratefully acknowledged. The fieldwork in Kongsfjorden in May 2000 and 2001 was financed by the Norwegian Radiation Protection Authority; financial support was also provided by the marine surveillance program (Norwegian Environmental Ministry). This work was presented at the conference 2001: An Ocean Odyssey, 21–28 October, 2001, Mar del Plata, Argentina.

## References

1. AMAP (1998) Radioactivity. In AMAP Assessment Report: Arctic Pollution Issues. Arctic Monitoring and Assessment Programme (AMAP), Oslo. P. Strand et al., Eds. pp. 525–619.

2. Brown, J.E., Kolstad, A.K., Brungot, A.L., Lind, B., Rudjord, A.L., Strand, P., and Føyn, L. (1999) Levels of $^{99}$Tc in seawater and biota samples from Norwegian coastal waters and adjacent seas. *Mar. Pollut. Bull.* **38**, 560–571.
3. Brown, J.E., Iosjpe, M., Kolstad, K., Lind, B., Rudjord, A., and Strand, P. Temporal trends for $^{99}$Tc in Norwegian coastal environments and spatial distribution in the Barents Sea. *J. Environ. Radioact*., in press.
4. Gerland, S., Lind, B., Dowdall, M., Kolstad, A., and Brungot, A. Radionuclides in the Kongsfjorden area, Svalbard. In Proceedings of the International Conference on the Radioecology-Ecotoxicology of Terrestrial and Estuarine Environments, Aix-en-Provence, France, September 2001. Bréchignac, F., Ed. Radioprotection Colloquium Series, EDP Sciences, Paris, in press.
5. Smith, V., Ryan, R.W., Pollard, D., Mitchell, P.I., and Ryan, T.P. (1997) Temporal and geographical distributions of $^{99}$Tc in inshore waters around Ireland following increased discharges from Sellafield. *Radioprotection–Colloques* **32**, 71–77.
6. Dahlgaard, H. (1994) Sources of $^{137}$Cs, $^{90}$Sr, and $^{99}$Tc in the East Greenland Current. *J. Environ. Radioact.* **25**, 37–55.
7. Hop, H., Pearson, T., Hegseth, E.N., Kovacs, K.M., Wiencke, C., Kwasniewski, S., Eiane, K., Mehlum, F., Gulliksen, B., Wlodarska-Kowalczuk, M., Lydersen, C., Weslawski, J.M., Cochrane, S., Gabrielsen, G.W., Leakey, R.J.G., Lønne, O.J., Zajaczkowski, M., Falk-Petersen, S., Kendall, M., Wängberg, S.-Å., Bischof, K., Voronkov, A. Yu., Kovaltchouk, N.A., Wiktor, J., Polterman, M., di Prisco, G., Papucci, C., and Gerland, S. The marine ecosystem of Kongsfjorden, Svalbard. *Polar Res*., in press.
8. Svendsen, H., Beszczynska-Møller, A., Hagen, J.O., Lefauconnier, B., Tverberg, V., Gerland, S., Ørbæk, J.B., Bischof, K., Papucci, C., Zajaczkowski, M., Azzolini, R., Bruland, O., Wiencke, C., Winther, J-G., and Dallmann, W. The physical environment of Kongsfjorden-Krossfjorden, an Arctic fiord system in Svalbard. *Polar Res*., in press.
9. Dahlgaard, H. (1995) Transfer of European coastal pollution to the Arctic: radioactive tracers. *Mar. Pollut. Bull.* **31**, 3–7.
10. Smith, J., Ellis, K., and Jones, E. (1990) Cesium 137 transport into the Arctic Ocean through Fram Strait. *J. Geophys. Res.* **95**, 1693–1701.
11. Haugan, P. (1999) Structure and heat content of the West Spitsbergen Current. *Polar Res.* **18**, 183–188.
12. Gerland, S. and Grøttheim, S. (2001) Transport of artificial radionuclides with ocean currents, sea ice and particulate matter. In The Expeditions ARKTIS XVI/1 and ARKTIS XVI/2 of the Research Vessel Polarstern in 2000. Krause, G. and Schauer, U., Eds. *Rep. Polar Mar. Res.* **389**, 61–63.
13. Krause, G. and Schauer, U., Eds. (2001) The Expeditions ARKTIS XVI/1 and ARKTIS XVI/2 of the Research Vessel *Polarstern* in 2000. *Rep. Polar Mar. Res.* **389**, 108 p.
14. Kolstad, A.K. and Rudjord, A.L. (2000) Radioactive pollution in the marine environment—99Tc in seawater and biota in 1998 and 1999. *Strålevern Rapport* **2000:7**, 17 p. (in Norwegian).
15. Kolstad, A.K., Lind, B., and Rudjord, A.L. (1999) Technetium-99 analysis—Calibration routines and method description. *Strålevern Rapport,* 31 p. (in Norwegian).
16. Chen, Q., Aarkrog, A., Nielsen, S., Dahlgaard, H., Lind, B., Kolstad, A., and Yu, Y. (2001) Procedures for determination of 239, 240 Pu, 241 Am, 237 Np, 234, 238 U, 228, 230, 232Th, 99 Tc and 210 Pb–210 Po in environmental materials. *RISØ Report* **RisØ-R-1263(EN)**, 33 p.
17. Grøttheim, S. (2000) Artificial radionuclides in the northern European marine environment. *Strålevernrapport, Norwegian Radiation Protection Authority, Østerås, Norway* **2000:1**, 102 p.
18. Nies, H., Harms, I., Karcher, M., Dethleff, D., Bahe, C., Kuhlmann, G., Oberhuber, J., Backhaus, J., Kleine, E., Loewe, P., Matishov, D., Stepanov, A., and Vasiliev, O. (1998) Anthropogenic radioactivity in the Nordic seas and the Arctic Ocean—results of a joint project. *Dtsch. Hydrogr. Z.* **50**, 313–343.
19. Kershaw, P.J., McCubbin, D., and Leonard, K.S. (1999) Continuing contamination of north Atlantic and Arctic waters by Sellafield radionuclides. *Sci. Total Environ.* **238**, 119–132.
20. Ingvaldsen, R., Reitan, M., Svendsen, H., and Asplin, L. (2001) The upper layer circulation in Kongsfjorden and Krossfjorden—a complex fjord system on the west coast of Spitsbergen. *Mem. Natl. Inst. Polar Res., Special Iss.* **54**, 393–407.
21. Gerland, S. and Winther, J.-G. (2000) Development of thickness and temperature of first-year fast ice during freezing and melting. *Suppl. to Eos Trans. Am. Geophys. Union* **81**, F760.
22. Saloranta, T. and Svendsen, H. (2001) Across the Arctic front west of Spitsbergen: high-resolution CTD sections from 1998–2000. *Polar Res*. **20**, 177–184.
